# Effects of post-exercise cold-water immersion on performance and perceptive outcomes of competitive adolescent swimmers

**DOI:** 10.1007/s00421-024-05462-x

**Published:** 2024-03-28

**Authors:** Natanael P. Batista, Flávia A. de Carvalho, Caio R. D. Rodrigues, Jéssica K. Micheletti, Aryane F. Machado, Carlos M. Pastre

**Affiliations:** 1 Department of Physiotherapy, School of Technology and Sciences, Sao Paulo State University (UNESP), Presidente Prudente, Sao Paulo Brazil; 2 School of Exercise and Rehabilitation Sciences, The University of Toledo, 2801 Bancroft St, Toledo, OH 43606 USA

**Keywords:** Recovery, Swimming, Cryotherapy, Athletic performance, Adolescent

## Abstract

**Purpose:**

To evaluate the effects of repeated use of cold-water immersion (CWI) during a training week on performance and perceptive outcomes in competitive adolescent swimmers.

**Methods:**

This randomized-crossover study included 20 athletes, who received each intervention [CWI (14 ± 1 °C), thermoneutral water immersion (TWI) (27 ± 1 °C) as placebo, and passive recovery (PAS)] three times a week between the land-based resistance training and swim training. The interventions were performed in a randomized order with a 1-week wash-out period. We tested athletes before and after each intervention week regarding swim (100 m freestyle sprints) and functional performance (flexibility, upper and lower body power, and shoulder proprioception). We monitored athlete’s perceptions (well-being, heaviness, tiredness, discomfort and pain) during testing sessions using a 5-item questionnaire. Athlete preferences regarding the interventions were assessed at the end of the study. We used generalized linear mixed models and generalized estimating equations for continuous and categorical variables, respectively (intervention x time).

**Results:**

We found a time effect for swim performance (*p* = .01) in which, regardless the intervention, all athletes improved sprint time at post-intervention compared to baseline. There was an intervention effect for pain (*p* = .04) and tiredness (*p* = .04), but with no significant post-hoc comparisons. We found no significant effects for other outcomes. All athletes reported a preference for CWI or TWI in relation to PAS.

**Conclusion:**

The repeated use of CWI throughout a training week did not impact functional or swim performance outcomes of competitive adolescent swimmers. Perceptive outcomes were also similar across interventions; however, athletes indicated a preference for both CWI and TWI.

**Supplementary Information:**

The online version contains supplementary material available at 10.1007/s00421-024-05462-x.

## Introduction

There is increased demand in the competitive swimming field to explore aspects related to training biomechanics and physiology for performance enhancement (Crowley et al. 2017). Resistance training is commonly practiced before swimming training sessions and has been recommended to be included as part of training routine instead of high volume of swimming training alone (Crowley et al. 2017). Strength training or swimming-specific dry-land training programs have shown benefits in enhancing swimming performance (Girold et al. 2007; Aspenes and Karlsen 2012; Arsoniadis et al. 2022), but they also result in increased rating of perceived exertion (RPE) and acute physiological and biomechanical alterations (Arsoniadis et al. 2019). Therefore, researchers have been investigating the stress-recovery balance mainly by means of post-exercise recovery strategies aiming at the return of metabolic and neuromuscular function (Kellmann et al. 2018).

In this scenario, cold-water immersion (CWI) stands out because of its relatively low cost and easy applicability. The implementation of post-exercise CWI between dry-land resistance training and swimming training can be the key to this stress-recovery balance, since it has been suggested that CWI may improve subsequent training load and quality (Barnett 2006; Kellmann 2010). Previous studies have extensively researched CWI acute responses (Chaillou et al. 2022) and found significant improvements in delayed-onset muscle soreness (DOMS) (Batista et al. 2023) and general RPE (Hohenauer et al. 2015). However, CWI has been frequently used by coaches and athletes regardless of physiological and scientific rationale (Allan et al. 2021). Considering that the period between resistance and swimming training needs to be adequate to facilitate performance improvements (Arsoniadis et al. 2022), CWI could be an asset to enhance swimmer’s recovery.

Since athletes are constantly exposed to high training loads, researchers are currently interested in the effects of repeated use of CWI (Ihsan et al. 2021; Tavares et al. 2020, 2019; Chaillou et al. 2022) and its possible detrimental effects on training adaptations (e.g., decreased functional performance, and impaired hypertrophy), mainly because its effects on exercise-induced muscle damage is still controversial (Broatch et al. 2018). However, studies found no harm to performance when CWI was applied repeatedly (Tavares et al. 2020; Halson et al. 2014; Rowsell et al. 2011). In fact, recent research suggests that CWI may actually improve endurance performance, and discrepancy between findings may be found because different types of training (e.g., resistance, endurance and sprint exercises) may be affected differently (Chaillou et al. 2022; Malta et al. 2021; Ihsan et al. 2021). Although CWI may decrease swim performance immediately after its application (Parouty et al. 2010), 2 weeks of daily application have shown to improve sleep quality, reduce muscle soreness and promote faster return to homeostatic parasympathetic activity (Al Haddad et al. 2012). If CWI could improve swimmers’ perceptions of recovery from resisted exercises and, consequently, swimming training quality, we may be able to observe enhancements in different aspects of performance, such as power measured by functional tests and, ultimately, swim performance.

Researchers are recommended to use an integrated approach to evaluate post-exercise recovery (Kellmann et al. 2018; Micheletti et al. 2019). The evaluation of functional tests, for example, provides a more comprehensive understanding of the athletes’ overall physical capabilities (Smith et al. 2017) and enables the isolation of the specific effects of CWI on swimming performance. It also may offer valuable insights for coaches, professionals and athletes by identifying areas for improvement that extend beyond the scope of traditional swimming assessments. Moreover, because performance is the main goal in sport, perceptive outcomes are usually neglected. Due to the multifactorial nature of recovery, studies should investigate both physiological and psychological outcomes by integrating a method that explores different aspects of performance as well as perceptive variables (Kellmann et al. 2018; Micheletti et al. 2019). This approach has been previously used in swimmers and suggested to contribute to a better understanding of post-exercise recovery process (Carvalho et al. 2023). Therefore, our objective was to investigate the effects of repeated use of CWI, compared to thermoneutral water immersion (TWI) and passive recovery (PAS), on performance and perceptive outcomes of competitive adolescent swimmers. We hypothesized that CWI would improve both swimmers’ perceptions and performance, while PAS and TWI would not provide changes on any outcome.

## Methods

### Participants and study design

A convenience sample of 25 competitive adolescent swimmers from a local team was screened. To be included, male and female athletes should be healthy, over 12 years old and train regularly (i.e., 6 days/week) on the competitive team. After screening, 20 athletes were included in the study (sex: 12 male/8 female, age: 14.05 ± 1.79 years; mass: 59.86 ± 12.85 kg; height: 1.66 ± 0.10 m; BMI: 21.62 ± 3.07 kg/m^2^).

All procedures were registered on the Brazilian Registry of Clinical Trials (No. RBR-67qgm2) and approved by the Sao Paulo State University Research Ethics Committee (CAAE: 92352318.0.0000.5402). Both participants and their legal guardians were informed about the study’s risks and benefits and given informed consent.

### Procedures

This study is a randomized-crossover placebo-controlled clinical trial with 1:1:1 allocation reported according to the consolidated standards of reporting trials (CONSORT) checklist for crossover trials (Schulz et al. 2010). The study was conducted over 6 weeks of the swimmers regular training which was composed of a resistance land-based training followed by swimming training during weekdays (i.e., Monday through Friday), and swimming tournament simulation on Saturdays (testing session). The land-based resistance training (37.8 ± 4.5 min) was already part of the team’s training plan and was performed every weekday at the beginning of each session before swimming training. This land-based resistance training was an adaptation of Morais et al. (2018) training protocol which consisted of a warm-up (e.g., 5 min of light run) followed by three sets of sit-ups, push-ups, squats, vertical jumps, burpees and mountain climbers and then, elastic band shoulder exercises (e.g., flexion, extension, abduction, internal and external shoulder rotation). The swimming training (136.8 ± 18.7 min; water temperature: 26 ± 2 °C) followed the periodization prescribed by the coach, and consisted of four sections: warm-up, skills sets, main sets and cool-down. A 15 min interval separated land-based and swimming training.

During the testing sessions (i.e., Saturdays), we assessed swim and functional performance. Athletes completed a standard warm-up followed by two 100 m freestyle sprints, (with a 30 min interval between them, and a standard cool-down) and a battery of functional performance tests.

**Fig. 1:**
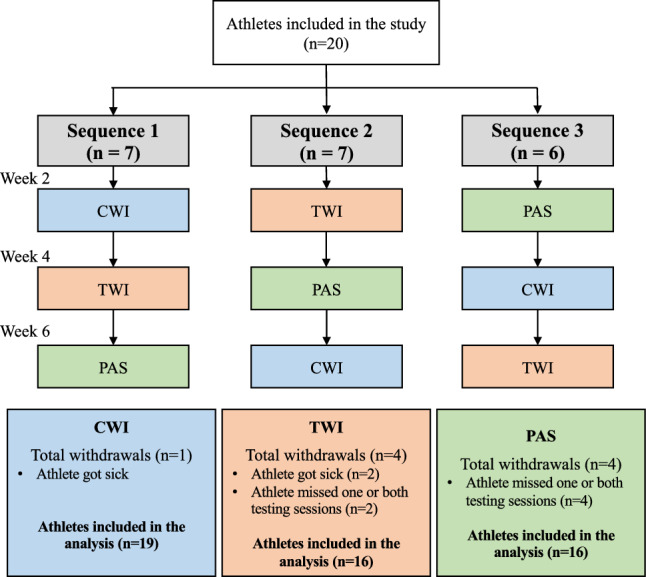
Randomization flowchart and sample size for each intervention at the end of the study (*CWI* cold-water immersion, *TWI* thermoneutral water immersion, *PAS* passive recovery)

Prior to the study, we performed a blinded balanced-block randomization with a balanced sex, age and competitive level ratio, and allocated athletes so that they performed all interventions (PAS, TWI and CWI) under three different sequences (Fig. 1). Aiming to allow baseline comparisons, the participants did not perform any intervention on odd weeks, and we implemented interventions on even weeks. Therefore, the study was six weeks in duration, classified as baseline weeks (week 1, week 3 and week 5) and intervention weeks (week 2, week 4 and week 6) (Fig. 1). On baseline weeks, participants trained as usual during weekdays without receiving any kind of intervention and were assessed on the respective Saturday to obtain reference values. For intervention weeks (i.e., week following baseline week), participants trained as usual and were assigned to receive one of the three recovery interventions, and then re-assessed on the respective Saturday. The option of having a baseline week prior to an intervention week was taken aiming to avoid possible confounding effects of the previous intervention.

To guarantee concealed allocation, an independent researcher assigned the participants, thus, therapists and participants had no previous knowledge of the intervention sequence. The assessors were also blinded to intervention allocation. All participants were analyzed for all three interventions, regardless of intervention adherence; however, participants were excluded from the analysis of the respective intervention if they missed one or both testing sessions.

### Interventions

**Fig. 2:**
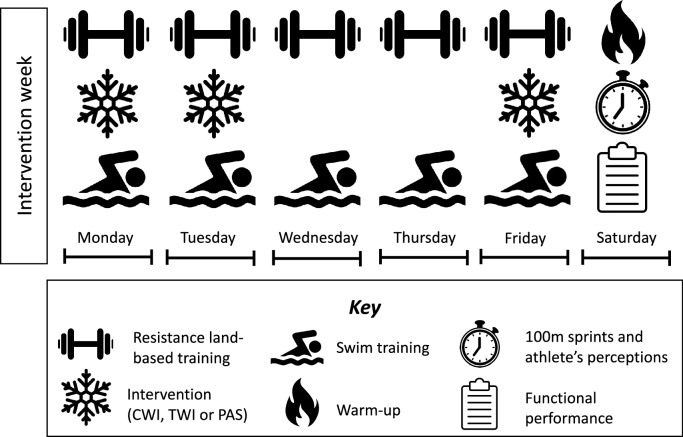
Study procedures during an intervention week (*CWI* Cold-water immersion, *TWI* thermoneutral water immersion, *PAS* passive recovery)

We implemented interventions on 3 days of the intervention week (Mondays, Tuesdays, and Fridays) immediately after the land-based resistance training and before swimming training (Fig. 2).

We instructed athletes assigned to the PAS to rest for 12 min, but were free to sit, stand or walk by the pool without entering the water or engaging in physical activity to simulate the actual training scenario.

We instructed athletes assigned to the TWI to enter a standard 200 L water tank, with thermoneutral water (27 ± 1° C) at the shoulder level for 12 min. We adopted the strategy reported by Broatch et al. (2014) by adding a skin cleanser solution (Cetaphil, Gentle Skin Cleanser, Australia) to the water in plain sight and leading participants to believe that this intervention was beneficial for post-exercise recovery. In our study, TWI was considered our placebo intervention.

We instructed athletes assigned to the CWI to enter a standard 200 L water tank with cold water (14 ± 1° C) at the shoulder level for 12 min. We monitored water temperature using a thermometer (XT-1234, Xtrad/Knup, Brazil) and controlled temperature by adding ice cubes to the tank. The dose was based on evidence for recovery of muscle soreness (11–15° C for 11–15 min) (Batista et al. 2023) and autonomic activity (14 ± 1° C) (Bastos et al. 2012). The water was constantly agitated by the therapist aiming to avoid a warmed thermal layer around the body during immersion.

### Outcomes

The study was conducted during the preparation training phase (pre-season). We monitored external training load by weekly training volume (distance swam in meters), that was previously stablished by the coach (Supplementary file 1). We monitored internal training load by multiplying the Session Rating of Perceived Exertion (sRPE; 0–10 Borg scale) (Borg 1982) in both resistance (assessed immediately after) and swimming training (assessed 20–30 min after the main sets) by the session duration in minutes. Weekly RPE (wRPE) was obtained by the sum of resistance and swimming sRPE of all weekdays and represented the athlete’s internal training load.

We monitored the participant’s perceptions using a Likert scale from 1 to 5 (nothing, a little, moderate, a lot and extremely) regarding athlete’s well-being, heaviness, tiredness, discomfort and pain. Participants were previously familiarized with this scale throughout a pilot study. We asked participants to rate their perceptions after the 100 m sprint regarding the following sprint phases: beginning of sprint, middle of sprint, end of sprint, and after sprint. We assessed participant’s perceptions only in the intervention week. At the end of the study, all athletes were asked regarding their preference between the three interventions with the following question: “With which intervention did you feel more recovered for the swimming training?”.

We obtained sprint times from the coach with digital chronometer (HS-3, Casio, USA) during two 100 m freestyle sprints in a 25 m pool, with a 30 min interval between them. The best sprint (i.e., lowest time) and its respective data (i.e., perceptions) were considered for analysis. For the purpose of this study, we adjusted an international scoring system, known as FINA points, to individual’s performance using the participant’s best performance from the latest season instead of the modality’s world record (http://www.fina.org/content/fina-points). This outcome varies from 0 to 1000 and was calculated as FINA Points = $1000 *({B/T)}^{3}$, where *B* is the participant’s best performance and *T* is the sprint time.

We assessed flexibility using the sit-and-reach test (Muyor et al. 2014). Participants were asked to sit with their feet touching the testing box and then slide with the dominant hand on top of the other at maximum distance without flexing their knees. The best score in centimeters of three attempts with 30 s interval was collected for analysis.

We assessed lower limb power using the squat jump test (Markovic et al. 2004). Participants were required to keep the soles of their feet in contact with the jump platform (Multisprint, Hidrofit, Brazil), lower limbs flexed at 90°, hands on the waist, trunk erect and without previous movements. The participants were then required to jump, keeping their knees extended until they touched the platform again. The best score in centimeters of three attempts with 30 s interval was collected for analysis.

We assessed upper limb power by bench press and pull up movements using a linear velocity transducer (T-force System, Ergotech, Murcia, Spain) (Pérez-Castilla et al. 2019). The tests were performed according to the equipment instructions (http://www.tforcesystem.com/tutorial.php) and their reliability was previously established in a pilot test. For the bench press, the participants laid on a bench with both feet on the ground. The transducer was attached to a free bar with fixed weight of 10 kg and the participants were asked to lower the bar slowly to the chest and then press to full arms’ extension explosively keeping their head, shoulders, and buttocks in contact with the bench. For the pull up, participants started from a neutral position, standing on the ground or a bench, depending on the participant’s height, with the arms extended and the transducer attached to their waist. Participants were then asked to raise themselves explosively. Participants completed three trials for each movement, with 30 s interval, and the best mean propulsive velocity in m/s was considered for analysis.

We assessed shoulder proprioception by an adaptation of the laser-pointer assisted angle reproduction test (Balke et al. 2011). The reliability of this test was previously established in a pilot test. A laser-pointer was fixed below the deltoid muscle of the participant’s dominant arm with a Velcro strap, and they were asked to stand, with bathing suits, at 1 m from a board fixed on the opposite wall with three targets marked individually at 55°, 90° and 125° of shoulder flexion. Initially, the participant was required to point the laser on the targets and memorize the three joint positions and then reproduce them in a randomized order but with their eyes covered. The points where the laser was aimed on the board were marked by the investigator without informing the patient. The distance in cm from the target on both vertical and horizontal axis were transformed in degrees by a custom software using the formula *d* = 100 x tan*Z* and the angular deviations were obtained $AD= \sqrt{{ZX}^{2}+ Z{Y}^{2}}$. The smaller angular deviation in degrees was registered for analysis.

### Statistical analysis

Statistical analyses were performed on SPSS software version 18 (SPSS Inc. Chicago, EUA). We used repeated measures analysis of variance (ANOVA) to compare internal training loads (i.e., wRPE) between interventions and time. We analyzed effects of interventions on perceptive outcomes by Generalized Estimating Equations with ordinal distribution and cumulative logit link function. The predictors were intervention (PAS, CWI, and TWI) and time (Beginning of the sprint, Middle of the sprint, End of the sprint, after the sprint). Dependent variables were rated by a 5-point Likert scale and the last category (extremely) was used as reference. We analyzed effects of interventions on performance outcomes by Generalized Linear Mixed Models with normal distribution with intervention (PAS, CWI, and TWI) and time (baseline and post-intervention testing session) as predictors for flexibility, bench press, pull up and proprioception, and gamma distribution for sprint time and squat jump. Bonferroni adjustments were used for all significant main effects. Parameter estimates (*B*) and Exp (*B*) were reported along with 95% confidence intervals (CIs), descriptive data were reported as median with minimum and maximum values for categorical data and means with standard errors for continuous data. All analyses assumed level of significance of *p* < 0.05. The athletes’ preference regarding interventions was analyzed descriptively.

## Results

The analysis utilized data from 20 participants who completed all three interventions. However, due to missing data, the total number of participants considered for each intervention varied: 19 for CWI, 16 for TWI, and 16 for PAS. These dropouts were attributed to participants missing one or both testing sessions within specific conditions (Fig. 1).

We monitored the training load throughout the study due to the crossover design. Our repeated measures ANOVA of internal training loads found no significant main effects and, therefore, we can ensure that all interventions were performed under the same conditions (Supplementary file 2).

**Fig. 3:**
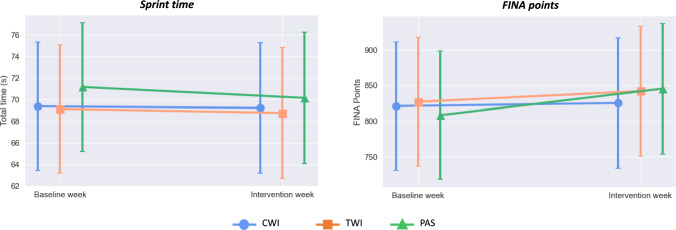
Swim performance measured by sprint time and FINA points during baseline and intervention weeks (*CWI* cold-water immersion, *TWI* thermoneutral water immersion, *PAS* passive recovery)

Table 1 presents the detailed results of interventions on performance outcomes. There were no interaction effects (intervention*time) for swim or functional performance outcomes. We only observed time effects for sprint time (*p* = 0.01) and FINA points (*p* = 0.01) of which, regardless the intervention, the athletes improved swim performance at post-intervention compared to baseline (Fig. 3). We found no significant main effects for any functional performance outcomes.

**Table 1:** Generalized linear models for performance outcomes after 1 week of intervention between resistance and swimming training

	Within-condition difference *B* (CI 95%)			Between-condition differences^a^ *B* (CI 95%)			
	PAS Mean ± SD	TWI Mean ± SD	CWI Mean ± SD	CWI–PAS		CWI–TWI	TWI–PAS
Functional performance outcomes							
Squat jump (cm)							
Baseline	26.07 ± 4.6	26.41 ± 4.6	26.91 ± 4.61	2.05(− 1.14 to 5.24)		0.69 (− 2.51 to 3.88)	1.36 (− 1.95 to 4.68)
At 1 wk	25.41 ± 4.72	26.78 ± 4.72	27.47 ± 4.74				
Baseline → 1 wk	− 0.66 (− 1.44 to 0.13)	0.37 (− 0.45 to 1.19)	0.55 (− 0.22 to 1.33)				
Flexibility (cm)							
Baseline	33.56 ± 7.2	34.33 ± 7.20	34.12 ± 7.18	0.95 (− 4.21 to 6.12)		− 1.08 (− 6.25 to 4.08)	2.04 (− 3.35 to 7.42)
At 1 wk	33.36 ± 7.68	35.40 ± 7.68	34.32 ± 7.66				
Baseline → 1 wk	− 0.20 (− 1.50 to 1.10)	1.07 (− 0.23 to 2.37)	0.20 (− 0.99 to 1.39)				
Bench press (m/s)							
Baseline	1.22 ± 0.24	1.25 ± 0.24	1.28 ± 0.26	0.04 (− 0.12 to 0.19)		0.09 (− 0.06 to 0.25)	− 0.06 (− 0.23 to 0.10)
At 1 wk	1.28 ± 0.24	1.21 ± 0.24	1.31 ± 0.28				
Baseline → 1 wk	0.06 (− 0.03 to 0.15)	− 0.04 (− 0.12 to 0.05)	0.03 (− 0.04 to 0.11)				
Pull up (m/s)				− 0.02 (− 0.23 to 0.18)		− 0.10 (− 0.30 to 0.11)	0.07 (− 0.14 to 0.29)
Baseline	0.34 ± 0.32	0.43 ± 0.32	0.48 ± 0.35				
At 1 wk	0.46 ± 0.32	0.53 ± 0.28	0.43 ± 0.30				
Baseline → 1 wk	0.11 (− 0.08 to 0.30)	0.10 (− 0.09 to 0.29)	− 0.05 (− 0.22 to 0.13)				
Proprioception at 55° (°)							
Baseline	10.82 ± 6.56	8.80 ± 6.56	10.59 ± 6.70	0.06 (− 4.66 to 4.78)		1.52 (− 3.21 to 6.24)	− 1.46 (− 6.38 to 3.46)
At 1wk	12.23 ± 7.00	10.77 ± 7.00	12.29 ± 7.00				
Baseline → 1 wk	1.41 (− 5.02 to 2.21)	1.97 (− 1.64 to 5.58)	1.69 (− 1.68 to 5.07)				
Proprioception at 90° (°)							
Baseline	7.69 ± 3.68	6.00 ± 3.68	6.87 ± 3.78	− 0.05 (− 2.21 to 2.12)		0.38 (− 1.78 to 2.55)	− 0.43 (− 2.69 to 1.82)
At 1wk	5.56 ± 3.20	5.13 ± 3.20	5.51 ± 3.22				
Baseline → 1 wk	− 2.13 (− 4.33 to 0.06)	− 0.88 (− 3.07 to 1.32)	− 1.36 (− 3.41 to 0.69)				
Proprioception at 125° (°)							
Baseline	8.87 ± 6.64	12.71 ± 6.64	11.49 ± 6.74	3.46 (− 0.96 to 7.88)		2.50 (− 1.93 to 6.92)	0.96 (− 3.65 to 5.57)
At 1 wk	9.03 ± 6.56	9.99 ± 6.56	12.49 ± 6.52				
Baseline → 1 wk	0.16 (− 2.95 to 3.28)	− 2.72 (− 5.83 to 0.40)	0.99 (− 1.92 to 3.92)				
Swim performance outcomes							
Total time (s)							
Baseline	71.17 ± 5.96	69.14 ± 5.96	69.39 ± 5.96	− 0.92 (− 5.01 to 3.17)		0.46 (− 3.63 to 4.55)	− 1.38 (− 2.88 to 5.64)
At 1 wk	70.16 ± 6.08	68.77 ± 6.08	69.24 ± 6.05				
Baseline → 1 wk	− 1.01 (− 1.74 to − 0.29)	− 0.37 (− 1.08 to 0.34)	− 0.16 (− 0.81 to 0.49)				
FINA points							
Baseline	808.34 ± 90.28	827.11 ± 90.28	821.14 ± 90.01	− 20.05 (− 95.61 to 55.52)		− 16.66 (− 85.13 to 51.81)	− 3.39 (− 68.76 to 61.99)
At 1 wk	845.43 ± 91.40	842.05 ± 91.40	825.39 ± 91.22				
Baseline → 1 wk	37.10 (11.41 to 62.79)	14.94 (− 10.75 to 40.63)	4.25 − 19.32 to 27.83)				

^a^Post-intervention *CI 95%* confidence interval 95%, *PAS* passive recovery, *TWI* thermoneutral water immersion, *CWI* cold-water immersion; *wk* week, *SD* standard deviation

**Fig. 4:**
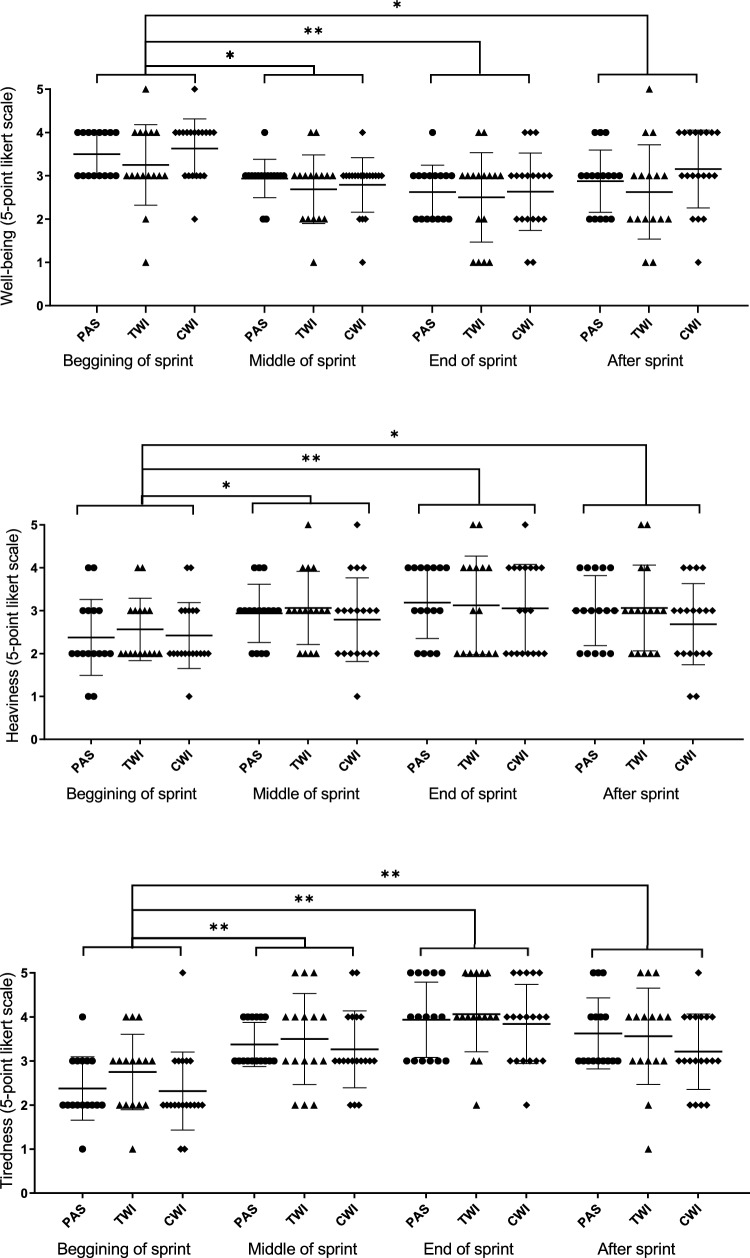

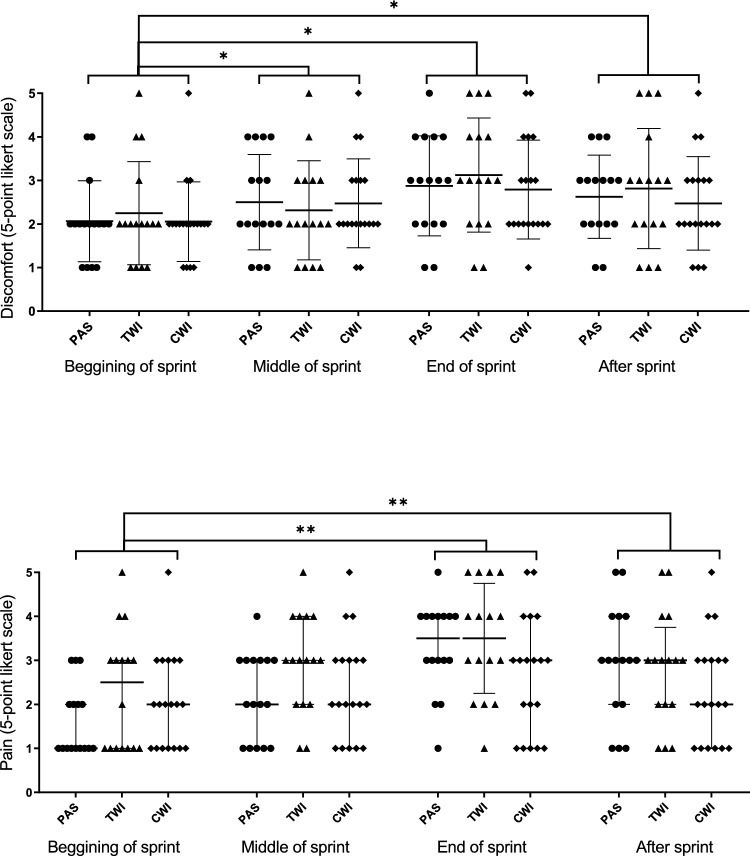
Swimmers’ perceptions during 100-m sprint stages. *Time effect post-hoc (*p* < .05), **Time effect post-hoc (*p* < .01) (*CWI* cold-water immersion, *TWI* thermoneutral water immersion, *PAS* passive recovery)

Figure 4 presents the results of swimmers’ perceptions regarding the 100-m sprint. As expected, there was a time effect (*p* < 0.01) for all perceptive outcomes in which the last stages of the sprint yielded the worst perceptions as observed by the increased chances of reporting pain, poor well-being, discomfort, tiredness and heaviness. There was a main intervention effect for pain (*p* = 0.04) and tiredness (*p* = 0.04); however, when looking at post-hoc comparisons, we observed no differences between interventions. No significant intervention*time interaction effects were observed.

After the trial, the participants were asked regarding their preference between the recovery interventions. Thirteen athletes (65%) felt more recovered after CWI, four athletes (20%) preferred TWI, and three athletes (15%) found both CWI and TWI effective. None of the athletes preferred passive recovery.

## Discussion

This study aimed to investigate the effects of repeated use of CWI in competitive adolescent swimmers compared to control and placebo. Our main finding showed that repeated use of CWI during a training week did not change functional (flexibility, shoulder proprioception, lower and upper body power) nor swim performance (100 m freestyle sprint time). We also found a main intervention effect for pain and tiredness during the sprint.

Considering training-recovery balance, the interest in the effects of repeated use of CWI has risen considerably in order to speed-up recovery and improves training quality (Ihsan et al. 2021). Our results did not support our initial hypothesis that the repeated use of CWI between resistance and swim training would improve swim performance. Nevertheless, our results also do not support the belief that CWI could impair training adaptations (Kwiecien and McHugh 2021; Yamane et al. 2006), as we observed improvements in swim performance (sprint time and FINA points) regardless of intervention. This finding corroborates previous studies that found similar improvements in cycling performance between repeated use of CWI and passive recovery (Aguiar et al. 2016; Halson et al. 2014; Vaile et al. 2008).

Similarly, we found no differences in functional performance outcomes (flexibility, power, and proprioception) among interventions. The evaluation of these variables provides a more nuanced and detailed analysis of the impact of CWI on various aspects of athletic performance. A systematic review found that the repeated use of CWI decreased performance gains for 1RM (one-maximal repetition), maximal isometric strength and endurance (Malta et al. 2021), parameters which were not assessed in our study. In addition, it is worth noting that the duration of the CWI protocols in the studies included in this analysis ranged from four to 12 weeks. Other studies, applying CWI for 3 weeks, reported favorable effects of CWI on countermovement jump performance in volleyball (Tavares et al. 2020) and rugby (Tavares et al. 2019) athletes. Given these findings, it appears that a one-week evaluation may not provide sufficient time to observe changes in these functional parameters and, therefore, future studies assessing the effects of long-term CWI on swimmers are needed.

It is also important to emphasize that a neutral result should not be underestimated and, perhaps, the benefit of the cold is elsewhere (e.g., perception outcomes). Ihsan et al. (Ihsan et al. 2021) support the current findings highlighting that, when time to recovery is limited, CWI can improve training performance which might outweigh possible decrements to hypertrophy, especially when hypertrophy is not the main goal. For instance, our examination of perceptive outcomes during sprints revealed main intervention effects for pain and tiredness without significant post-hoc comparisons, which usually happens due to lack of statistical power. Although not significant, a trend for reduction of pain over the last strokes was observed favoring CWI in relation to PAS (*p* = 0.05). We believe that the repetitive use of CWI may help athletes feel more tolerant to pain, given its documented advantages in alleviating muscle soreness (Batista et al. 2023) and increasing pain threshold (Klich et al. 2018). These benefits could potentially be enhanced with a repeated application protocol. As training progresses and symptoms become more frequent, the impact of CWI could potentially become more pronounced.

Interestingly, pain perception did not differ between CWI and TWI. In the present study, we led participants to believe that TWI with the skin cleanser solution would be beneficial for post-exercise recovery. A placebo intervention can elicit expectations and promote health benefits via brain-body responses, thereby, contributing to perception of recovery (Wager and Atlas 2015). This was confirmed by the preference for TWI expressed by 20% of the participants. In fact, some athletes might not tolerate CWI protocols which can affect post-training experiences and, therefore, their opinion should be considered during the decision making of interventions to be used.

Some researchers discuss the appropriateness of the use of CWI in sight of its modest effect sizes (Wilson et al. 2021). One of the aims of recovery strategy is to improve the athlete’s experience between training stimuli so they can be recovered not only physically but also psychologically, by promoting general well-being. CWI is known to anticipate metabolic and autonomic recovery after exhaustive exercise (Bastos et al. 2012) in addition to reduce fatigue and swelling, which may alleviate perceptions of discomfort, thereby allowing participants to perform better during training sessions (Roberts et al. 2014). Despite our results not affirming this rationale through the absence of improvements in performance outcomes, it gains support from the high acceptance rate among swimmers, as none favored passive recovery, while 65% expressed a preference for cold-water immersion.

Some strengths and limitations of the present study should be addressed. The crossover design can be considered as a strength, since it allowed assessing the same sample in different conditions/interventions, but it restricted the intervention duration to a single week. Although it was enough time to observe performance enhancements in response to training, it may not be sufficient to study differences between experimental conditions. The study included individuals from 12 to 20 years old which may be a heterogeneous sample considering hormonal differences and, therefore, these results may not be extrapolated to the adult population. However, considering that this age range is a decisive period for preparation to elite competition we believe that our results may help coaches to improve adolescent swimmers training. Although we provided participants with instructions, we could not guarantee they gave their best when performing performance tests or rating their perceptions. Finally, swimmers could be conditioned to water immersion, which possibly influenced our findings and, therefore, this should be considered when extrapolating these results to other sports.

## Practical applications

The debate surrounding the implementation of post-exercise recovery programs for competitive athletes persists, with the objective of optimizing their performance. Although CWI is a commonly used post-exercise recovery intervention, studies in the literature present inconclusive results regarding its effectiveness, especially because training characteristics are not considered when implementing it. When applied during training sessions, we did not observe detriments in performance, and athletes felt more recovered after doing CWI or TWI, in comparison to PAS (no intervention), emphasizing the importance of recovery in training routines.

## Conclusion

In summary, we found that the repeated use of CWI throughout a training week did not impact functional or swim performance outcomes of competitive adolescent swimmers. Although perceptive outcomes during sprinting were also similar across interventions, athletes indicated a preference for both CWI and TWI in relation to PAS. Professionals and coaches may consider incorporating CWI as a recovery intervention for competitive adolescent swimmers during training weeks, with a focus on enhancing recovery rather than explicitly aiming for performance improvements. However, it is imperative to consider the athletes’ preferences and tolerance when incorporating CWI into their recovery protocols.

### Supplementary Information

Below is the link to the electronic supplementary material.

Supplementary file1 (PDF 58 KB)

Supplementary file2 (PDF 62 KB)

## Data Availability

The data that support the findings of this study are available from the corresponding author, NPB, upon reasonable request.

## Abbreviations

ANOVA: Analysis of variance
CIs: Confidence intervals
CWI: Cold-water immersion
DOMS: Delayed-onset muscle soreness
PAS: Passive recovery
sRPE: Session rating of perceived exertion
TWI: Thermoneutral water immersion
wRPE: Weekly rating of perceived exertion
