# Supplementary figures and images for: Effects of post-exercise cold-water immersion on performance and perceptive outcomes of competitive adolescent swimmers
Source: Eur J Appl Physiol. 2024 Mar 28;124(8):2439–50. doi: 10.1007/s00421-024-05462-x (PMC11322250; doi:10.1007/s00421-024-05462-x)

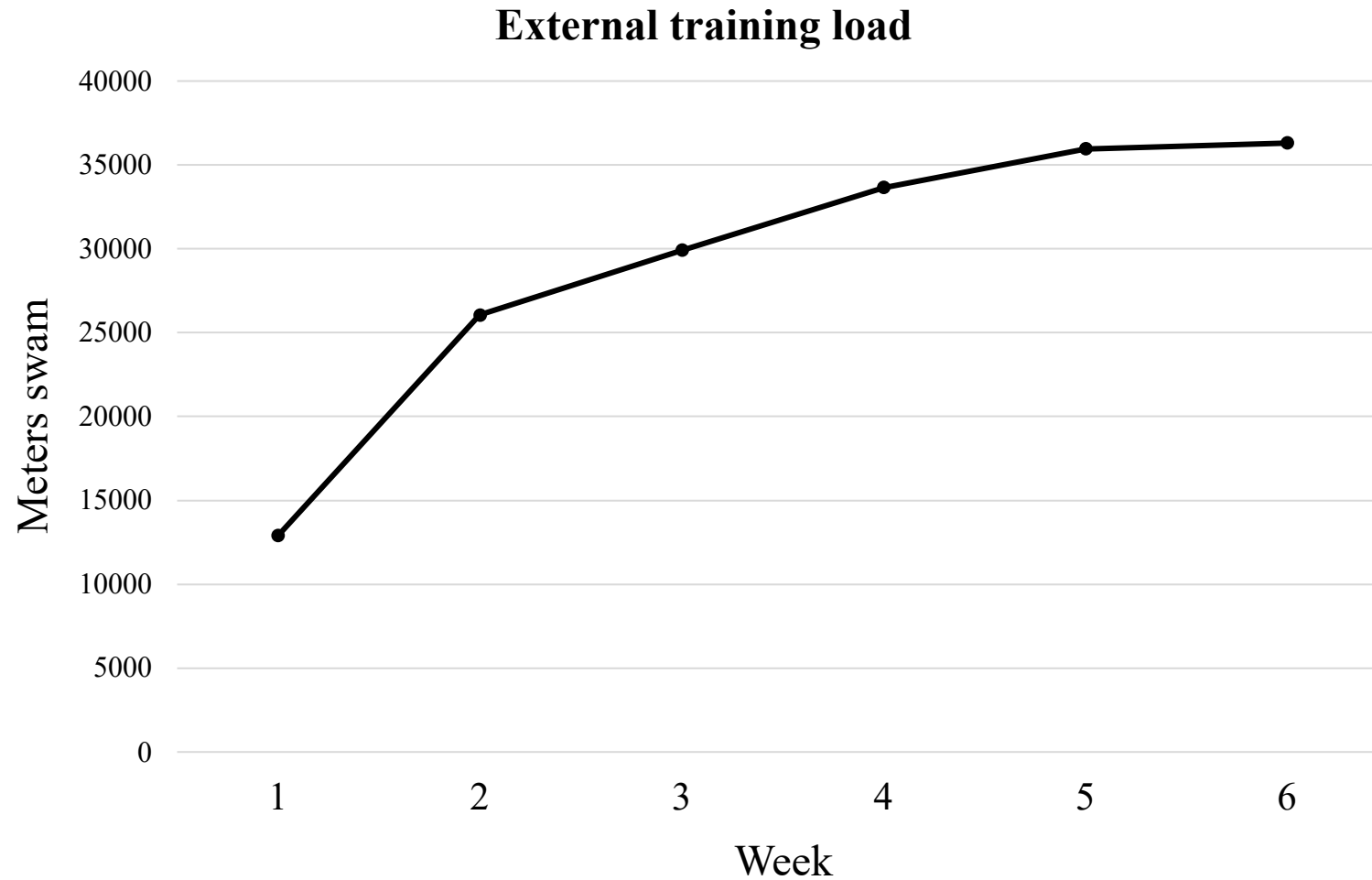

**Supplementary file 1.** Weekly external training loads.

Supplement: Supplementary file 1 — Supplementary file1 (PDF 58 KB) [file 421_2024_5462_MOESM1_ESM.pdf]
