# Supplementary material for: Effects of post-exercise cold-water immersion on performance and perceptive outcomes of competitive adolescent swimmers
Source: Eur J Appl Physiol. 2024 Mar 28;124(8):2439–50. doi: 10.1007/s00421-024-05462-x (PMC11322250; doi:10.1007/s00421-024-05462-x)
Supplement: Supplementary file 2 — Supplementary file2 (PDF 62 KB) [file 421_2024_5462_MOESM2_ESM.pdf]

Supplementary file 2 Repeated measures analysis of variance (ANOVA) comparing weekly rating of perceived exertion (wRPE) between interventions.

**CWI**		**TWI**		**PAS**		**ANOVA**		
Baseline week	Intervention week	Baseline week	Intervention week	Baseline week	Intervention week	**Intervention**	**Time**	**Interaction**
4642.8 ± 1371.1	4415.6 ± 941.0	4661.1 ± 1505.6	5061.8 ± 1067.0	4477.8 ± 1473.4	4829.3 ± 1256.5	p = .634	p = .419	p = .401

CWI Cold-water immersion, *TWI* thermoneutral water immersion, *PAS* passive recovery
